# Role of Perturbations of Epigenetic Processes in Cardiac Hypertrophy and Fibrotic Scarring

**DOI:** 10.2174/011573403X354902250708134633

**Published:** 2025-07-14

**Authors:** Brijesh Kumar Duvey, Devkant Sharma, Vishnu Mittal, Anjali Sharma

**Affiliations:** 1 Department of Pharmacology, Ch. Devi Lal College of Pharmacy, Jagadhari 135003, Haryana, India;; 2 Department of Pharmaceutics, Guru Gobind Singh College of Pharmacy, Yamunanagar 135001, Haryana, India

**Keywords:** Perturbations, epigenetic, cardiac hypertrophy, fibrotic scarring, histone modifications, chromatin remodeling

## Abstract

**Introduction:**

Cardiac hypertrophy and fibrotic scarring are fundamental contributors to the progression of heart failure and are associated with poor clinical outcomes. Recent advancements in cardiovascular research have emphasized the central role of epigenetic mechanisms, including DNA methylation, histone modifications, chromatin remodeling, and non-coding RNAs, in regulating the gene expression changes underlying these pathological processes.

**Methods:**

A comprehensive literature review was conducted using databases, including PubMed, Scopus, and Web of Science. Predefined keywords and inclusion/exclusion criteria were applied to select relevant studies focusing on epigenetic regulation in cardiac hypertrophy and fibrosis. Particular attention was given to studies involving DNA methyltransferases, TET enzymes, histone deacetylases, demethylases, chromatin remodeling complexes, and non-coding RNAs. Methodological transparency was ensured through a structured screening and data extraction process.

**Results:**

The review highlights the dynamic regulation of cardiac gene expression by epigenetic factors. DNA methylation and demethylation influence fibroblast activation and extracellular matrix deposition. Histone-modifying enzymes reshape chromatin architecture, altering transcriptional accessibility. Chromatin remodeling complexes regulate nucleosome positioning during stress responses. Emerging insights into epigenetic memory and transgenerational epigenetic inheritance further reveal the heritable nature of disease susceptibility.

**Discussion:**

These epigenetic perturbations collectively orchestrate the maladaptive gene expression patterns seen in cardiac hypertrophy and fibrosis. Understanding their roles provides a mechanistic basis for identifying biomarkers and therapeutic targets. The review also discusses recent omics-based technologies that aid in the characterization of epigenetic alterations, thereby expanding diagnostic and therapeutic horizons.

**Conclusion:**

Epigenetic mechanisms are pivotal in the development and progression of cardiac hypertrophy and fibrosis. Advances in epigenomic profiling are facilitating the development of precise and targeted interventions. This review underscores the potential of epigenetic therapies and calls for intensified research efforts to translate these findings into clinical applications.

## 1 INTRODUCTION

Cardiac hypertrophy and fibrotic scarring are hallmark pathological responses to stress or injury in the heart, often leading to heart failure if left unchecked [1]. Epigenetic processes, including DNA methylation, histone modifications, and non-coding RNA regulation, play a crucial role in orchestrating gene expression patterns that govern cardiac cellular responses. Perturbations in these epigenetic mechanisms can dysregulate the balance between adaptive and maladaptive remodeling, contributing to the progression of hypertrophy and fibrosis. Cardiac hypertrophy and fibrotic scarring are two significant heart conditions that can result from various stressors and pathological stimuli [2, 3]. These conditions often lead to heart failure if not addressed properly. Recent research has shown that epigenetic processes play an important role in the progression and development of these cardiac diseases. The aim of this review is to provide an understanding of how perturbations in epigenetic mechanisms contribute to cardiac hypertrophy and fibrotic scarring, highlighting key findings and future directions [4, 5]. DNA methylation is an epigenetic alteration that has been studied the most. This complex process consists of two main steps: methyl group addition and removal from DNA. The enzymes of the DNA methyltransferase (DNMT) family directly methylate cytosine bases in DNA. This mechanism is linked to gene silencing. For these molecules to work, a supply of methyl groups called S-adenosylmethionine (SAM) is required. Nevertheless, eliminating methyl groups from DNA is a difficult process that requires several enzyme reactions [6, 7]. Moreover, a large number of substances linked to DNA methylation have recently been identified [8-10]. These important molecules may be divided into categories based on the functions they perform, such as modifying the methylation process or introducing, eliminating, or preserving methyl groups on DNA. Other epigenetic processes that interact with the DNA methylation machinery include post-translational alterations brought about by histones and RNA-based mechanisms, such as miRNAs. This enables several epigenome components to influence numerous genes at once [11, 12].

An overview of the role played by DNA methyltransferases and 10-11 translocation enzymes in the dynamic control of DNA methylation patterns that affect extracellular matrix formation and fibroblast activation is given in the text. It examines how histone deacetylases and demethylases affect gene expression and chromatin structure, with a focus on how they affect cardiac remodeling. Furthermore, it emphasizes how important chromatin remodeling complexes are for modifying nucleosome accessibility and placement, which in turn controls transcriptional responses to stress. Additionally, it covers the effect of non-coding RNAs on post-transcriptional gene regulation in cardiac disease. To ensure methodological transparency and review credibility, relevant papers were evaluated, and data was gathered to summarize results on epigenetic regulation in fibroblast activation and cardiac remodeling.

### 1.1 Recent Advances in Epigenetic Modification

The two primary components of epigenetic modifications are controlled during the transcription of genes and the regulation of post-transcriptional genes. The former is used for maternal control, wherein external variables that affect children's altered gene expression include RNA editing, covalent histone modification, DNA methylation, chromatin remodeling, and gene silencing [13]. The focus of the latter is mostly on RNA control mechanisms, including riboswitches, antisense RNA, microRNA (miRNA), and non-coding RNA (ncRNA). Since only a small percentage of all coding genes are activated by somatic cells, epigenetic modifications are essential for regulating gene activation. These changes guarantee the preservation and generational transfer of genes required for a specific cell type [14, 15].

Epigenetic modifications constitute a potent but unpredictable regulatory mechanism in living organisms, serving as crucial supervisors of several critical processes and potential warning signals of disease [16, 17]. Finding and comprehending epigenetic modifications, however, is a difficult endeavor because of their complex nature and variable behavior. Many drawbacks plague the current methodologies used for epigenetic modification analysis, such as lengthy protocols, multiple steps, significant sample consumption, and limited sensitivity [18-24]. In order to accomplish signal amplification, they also combine high-performance nanomaterials, enzymes, or functionalized DNA, resulting in the construction of highly sensitive bio/chemical Nano-sensing surfaces [25]. Furthermore, technologies have facilitated rational research, enabling us to investigate the regulatory function of epigenetic alterations and the impact of exogenous compounds, such as medications, on epigenetic modifications [26]. The field of epigenetic study has expanded beyond genetics in recent years. Novel insights into the control of genes associated with human cardiovascular disease (CVD) are being provided *via* epigenetic mechanisms. For instance, aberrant DNA methylation has been seen in peripheral blood cells from AS patients as well as in tissues impacted by atherosclerosis (AS) [27, 28]. Heart injury is caused by acetylation of H3 and DNA demethylation on the p66Shc promoter, which is promoted by diabetes-related downregulation of SIRT1 and DNMT3b [29]. In type 2 diabetic monocytes, methyltransferase SETD7 generates H3K4 monomethylation, a distinct epigenetic signature [30, 31].

The insulin-like growth factor type 2 (IGF2) promoter, a crucial gene involved in regulating glucose homeostasis, cardiovascular health, and cholesterol metabolism, undergoes significant epigenetic changes during pregnancy. Notably, this promoter remains hypomethylated in pregnant women even six years post-gestation [32], indicating the long-lasting impact of pregnancy on maternal metabolic and cardiovascular regulation. Epigenetic technology may help us understand many human disorders and can be used to identify and select disease targets for early warning and prevention. The most prevalent kind of birth defect, congenital heart disease (CHD), is characterized by abnormalities of the heart wall, valves, or blood vessels. Numerous phenotypes may be further subdivided [33]. The results of the research demonstrated that patients with TOF had considerably higher levels of methylation in the RXRA promoter region of the right ventricular outflow tract myocardium. RXRA mRNA levels decreased along with this methylation shift, which might provide new information about the etiology of TOF and potential therapeutic targets [34, 35]. Modifications in histones are crucial for cardiac remodeling in diabetic cardiomyopathy (DbCM), a disorder that affects DM patients and is characterized by alterations in the morphology and physiology of the heart muscle [36]. Histone deacetylases (HDACs), which are targeted using non-specific inhibitors, have shown potential in diabetic mice by repairing cardiac remodeling brought on by diabetes mellitus, indicating that they may be used as DbCM therapeutic agents [37]. The disease known as hypertension, which is characterized by elevated blood pressure in systemic veins, has been examined using the epigenetic framework [38, 39]. The primary cause of coronary artery disease and brain disease is atherosclerosis, which is defined by the thickening of arterial walls [40-43].

A literature review was conducted to collect and synthesize current knowledge on the epigenetic regulation of cardiac hypertrophy and fibrosis. The search was performed across three major scientific databases: PubMed, Scopus, and Web of Science, using predefined keywords such as *“cardiac hypertrophy”, “fibrosis”, “epigenetics”, “DNA methylation”, “histone modification”, “chromatin remodeling”,* and *“non-coding RNAs”.* Articles published within the last 10 years were considered. Inclusion criteria focused on original research articles, reviews, and meta-analyses that addressed the roles of epigenetic mechanisms in cardiac remodeling. Studies not directly related to cardiac pathophysiology or lacking a clear epigenetic focus were excluded. Selected articles were screened by title and abstract, followed by full-text evaluation. Relevant findings were extracted, categorized by epigenetic mechanism, and critically analyzed to identify major regulatory pathways, emerging therapeutic targets, and areas that need further investigation. This approach ensured methodological transparency and the credibility of the review.

### 1.2 Epigenetic Modulation

A growing amount of evidence suggests that epigenetic regulation has a role in heart disease. Without changing the fundamental nucleotide sequence, fibroblast appearance and activity in cardiac fibrosis are most likely the result of modified transcription patterns of genetic material. Links between regulatory components and post-translational alterations need context-dependent investigation. This is especially crucial because of their physical contact with other proteins as well as their functional and regulatory linkages [44, 45].

### 1.3 Epigenetics in Cardiac Hypertrophy

Epigenetic regulation plays a crucial role in both cardiac hypertrophy and myocardial fibrosis, influencing gene expression through various mechanisms. Cardiac hypertrophy is a significant risk factor for cardiovascular disease, which encompasses heart failure, arrhythmias, and sudden death. Cardiac hypertrophy is related to both transcriptional reprogramming and embryonic gene expression, both of which are tightly regulated by epigenetic mechanisms. An increasing amount of research has demonstrated that epigenetics has a role in the occurrence and development of cardiac hypertrophy [46]. Epigenetic mechanisms have the ability to precisely regulate the expression of both embryonic and functional genes during cardiac hypertrophy, providing a large number of potential therapeutic targets [47]. Consequently, it is expected that one possible treatment strategy for cardiac hypertrophy will be epigenetic therapy. Cardiac hypertrophy and fibrotic scarring are hallmark pathological responses to stress or injury in the heart, often leading to heart failure if left unchecked [48]. Myocardial hypertrophy, characterized by an increase in the size of cardiomyocytes, typically occurs as an adaptive response to mechanical stress, such as hypertension or aortic stenosis. Epigenetic processes, including DNA methylation, histone modifications, and non-coding RNA regulation, play a pivotal role in orchestrating gene expression patterns in both hypertrophy and fibrosis [49]. For instance, the dysregulation of histone deacetylases (HDACs) and microRNAs, such as miR-208 and miR-133, contributes to cardiomyocyte enlargement and fibroblast activation [50, 51].

#### 1.3.1 Epigenetic Mechanisms in Cardiac Hypertrophy

##### 1.3.1.1 Histone Modifications

The acetylation and methylation of histones are critical in regulating gene expression during cardiac hypertrophy, affecting chromatin remodeling and transcriptional activity [51].

##### 1.3.1.2 Non-coding RNAs

MicroRNAs and long non-coding RNAs are emerging as significant regulators of hypertrophy, influencing gene expression and cellular responses.

##### 1.3.1.3 Genome-wide Analysis

Specific histone marks are associated with hypertrophy, revealing a complex network of enhancers and promoters that orchestrate hypertrophic gene expression [52, 53].

#### 1.3.2 Implications for Therapy

**Targeting Epigenetic Changes**: Novel therapeutic strategies are being developed to target epigenetic modifications, offering potential treatments for cardiac hypertrophy [54].

### 1.4 Epigenetics in Cardiac Fibrosis

A growing body of studies has shown in recent years the potential contribution of epigenetic pathways to the development and progression of CVDs. These mechanisms, which control gene expression independently of variations in the DNA sequence, include DNA methylation, nucleosome remodeling, and histone modifications [55-57]. The architecture of the chromatin is altered by these modifications, which impact the capacity of transcription factors to bind and regulate gene expression. Since epigenetic processes take effect soon after cardiac injury, they are the primary cellular mechanisms that regulate responses to environmental changes. Apart from cardiac fibrosis, their disruption has been directly associated with changes in organ and cellular functioning at the onset of several other illnesses, such as cancer [58]. Additionally, they are adaptable, which permits the creation of new therapeutic approaches. Epigenetic changes in CVDs are being studied more and more in an effort to develop novel anti-fibrotic medications. For example, it has been shown that DNA methylation is more prevalent in fibrotic hearts derived from human patients, and histone acetylation is a key regulatory mechanism governing cardiac fibrosis. By reversing these epigenetic markers, anti-fibrotic drugs may be developed [59, 60].

## 2 EPIGENETIC PROCESSES

The term “epigenetics” describes inherited changes in gene expression not brought about by differences in DNA coding. The two most studied epigenetic alterations are DNA methylation and histone modifications, which include acetylation, phosphorylation, ubiquitylation, and methylation. Two essential processes that collaborate to support chromatin remodeling are DNA methylation and histone modifications [61]. Thus, in higher eukaryotic cells, they aid in the variable control of gene expression. Furthermore, the newly identified regulatory non-coding RNAs (ncR NAs) have significant functions in the epigenetic environment. They cause post-transcriptional gene silencing and are effective in both pathological and non-pathological settings, such as neurological disorders, circulatory diseases, and cancer [62]. The following section will go into more detail on the precise methods and purposes of epigenetic alterations, emphasizing their vital roles in controlling gene expression and affecting a range of biological processes. Most often, these changes are overseen by:

### 2.1 DNA Methylation

The control of gene expression in the heart is known to depend on DNA methylation, and abnormal regulation of this process is linked to the development of cardiovascular diseases [63, 64]. DNA methylation and transcriptional silencing are connected. This epigenetic imprint is established by tetramethyl cytosine dioxygenases (TET) and removed by DNA methyltransferases (DNMT), two specific epigenetic mechanisms. Gene regulators often include CpG islands, which are repetitive areas of CpG sites that, when modified, stop transcription factors from binding DNA and thus obstructing gene translation [65]. DNMT3a and DNMT3b are primarily responsible for initiating *de novo* DNA methylation. DNMT3a plays a broader role, targeting various genomic regions and being more active in somatic cells, while DNMT3b is crucial for establishing methylation patterns during early embryogenesis, particularly in repetitive DNA sequences and imprinted genes. This functional distinction highlights their complementary roles in shaping the epigenome, while DNMT1 maintains these patterns going throughout DNA replication [66, 67]. However, TET enzymes directly counteract DNMT activity by removing DNA methylation, which raises gene expression [68-71]. Together with epigenetic changes, transcriptional regulatory effects are shown in Table **1**.

### 2.2 Modifications in Histone

Changes to the histone proteins that surround DNA, altering the shape of chromatin and the accessibility of genes. It has been determined that histone (H) regulators have a fibrogenic role in development of several CVDs. One important epigenetic mechanism regulating the expression of fibrotic genes is histone modification [71-74]. Histones pack DNA into chromatin, modify DNA accessibility by post-translational changes, and are important regulators of epigenetics. Modifications to histones occur near the N-terminal tail of histone proteins, at specific amino acids. These changes consist of monoubiquitylation, phosphorylation, acetylation, and methylation. The most extensive research has been done on these in connection with the onset of cardiac fibrosis, and they include methylation and acetylation, which will be covered in more depth below [75, 76].

### 2.3 Histone Methylation

Histone methylation may be linked to either active or inhibited gene transcription, depending on a number of variables including the specific amino acid modified, the degree, and the location of methylation on the histone tails. H3K4me1/2/3, H3K9me1, H3K27me1, H3K36me1/2/3, and H3k79me1/2/3 are active marks associated with gene activity, whilst the repressive marks H3K9me3, H3K27me3, and H4K20me2/3 [77] are related to gene silencing. Histone methylation is eliminated by histone lysine demethylases, or KDMs. Lysine specific demethylases (LSDs) and JMJC domain-containing family (JMJDs) are two categories into which they are often divided [78]. The two forms of histone methyltransferases (HMTs) that catalyze methylation (HMTs) are protein arginine methyltransferases (PRMTs) and protein lysine methyltransferases (PMTs) [79].

### 2.4 Histone Acetylation

The acetyl group imparts a negative charge that repels negatively charged DNA, histone acetylation is linked to active gene transcription [80]. Histone acetyltransferases (HATs) and histone deacetylases (HDACs), two competing enzyme families that add and remove acetyl groups, respectively, control the highly dynamic process of histone acetylation. Furthermore, the bromodomain and extra-terminal motif (BET) family of epigenetic proteins is known to aid in reading of histone acetylation marks [81, 82]. By binding to acetylated lysine residues in histone tails, these proteins function as reader proteins and attract other regulatory factors to initiate transcription [83]. Studies have shown that each of these epigenetic groups involved in histone acetylation has a major impact on the development of fibrotic disease [84].

### 2.5 Chromatin Remodeling

The process known as chromatin remodeling utilizes the energy produced during the breakdown of ATP to move, remove, deposit, or alter the structure of nucleosomes *via* chromatin remodeling complexes. By altering the shape of nucleosomes across the chromatin and, therefore, the accessibility of transcription factors and other DNA regulatory factors, this mechanism may regulate gene expression [85]. The 4 families of chromatin remodeling complexes are ISWI, CHD, SWI/SNF, and INO80 [86]. Their similar ATPase regions provide them with catalytic activity. These groups exhibit a range of characteristics. For example, nucleosome assembly and organization are improved, particularly after replication, by the ISWI and CHD subfamilies, which help with nucleosome assembly and distribution within DNA [87]. Through nucleosome sliding or removal at enhancers or promoters, SWI/SNF family regulates chromatin accessibility and impacts gene transcription [88]. Access remodelers often promote gene expression by opening chromatin and exposing transcription factor binding sites, while assembly remodelers suppress gene expression by forming densely packed nucleosome arrays.

Three main families of SWI/SNF chromatin remodeling complexes are now identified: non-canonical BAF, PBAF, and BAF. Finally, the INO80 family modifies nucleosomes without the need for replication by swapping out a particular histone with a conventional or variant histone [89]. While their early-stage impacts on CVDs have not garnered much study attention, chromatin remodelers play crucial roles in all stages of heart development [90]. Surprisingly, recent studies indicate that they may act as regulators that promote fibrosis. For instance, Brg1, a central ATPase of the SWI/SNF complex, has been linked to cardiac fibrosis, and CHD4, a component of the NuRD complex, has been shown to modulate fibrogenesis-related gene expression. For example, Pbrm1, a chromatin recognition component unique to PBAF, primarily regulates the TGFβ/BMP signaling cascade that causes mesenchymal cells to undergo osteogenic differentiation [91, 92].

The overgrowth, hardness, and/or scarring of various tissues are the hallmarks of fibrosis, which is brought on by the excessive deposition of extracellular matrix components like collagen. Recurrent infections, autoimmune reactions, allergic reactions, chemical attacks, radiation, and tissue injury are just a few of the causes that can result in persistent inflammatory reactions that eventually produce fibrosis [93, 94]. Even though current treatments for fibrotic diseases like idiopathic pulmonary fibrosis, liver cirrhosis, systemic sclerosis, progressive kidney disease, and cardiovascular fibrosis typically target the inflammatory response, there is mounting evidence that the mechanisms underlying fibrogenesis differ from those governing inflammation. In fact, several studies have shown that ongoing inflammation is not necessary to sustain developing and existing fibrosis [95]. The primary biological mediator of fibrosis is the myofibroblast, the primary cell that, when activated, produces collagen. Myofibroblasts are produced from a variety of sources, including resident mesenchymal cells, epithelial and endothelial cells, and circulating fibroblast-like cells called fibrocytes that are derived from bone-marrow stem cells [96]. These processes are referred to as the epithelial/endothelial-mesenchymal (EMT/EndMT) transition. Autocrine factors released by myofibroblasts, paracrine signals from macrophages and lymphocytes, and pathogen-associated molecular patterns (PAMPs) produced by pathogenic organisms that interact with the pattern recognition receptors (*i.e.*, TLRs) of fibroblasts are just a few of the many factors that can activate myofibroblasts. Cytokines (IL-13, IL-21, TGF-β1), chemokines (MCP-1, MIP-1β), angiogenic factors (VEGF), growth factors (PDGF) [97], peroxisome proliferator-activated receptors (PPARs), acute phase proteins (SAP), caspases, and components of the renin-angiotensin-aldosterone system (ANG II) are being investigated as potential targets for antifibrotic drugs because they are important regulators of fibrosis [98, 99].

Furthermore, it has been shown that Smarca4, a specific component of complex of the non-canonical BAF, has conflicting roles in the development and treatment of liver and lung fibrosis [100]. More investigation is required to completely understand the roles and processes of chromatin modification complexes in the development of fibrosis. Classical epigenetic alterations like acetylation and methylation undoubtedly impact the pathophysiology of cardiac fibrosis; however, the specific role of targeted epigenetic elements that control these processes remains unknown [101].

### 2.6 Non-coding RNAs

Small RNA molecules known as microRNAs (miRNAs) control gene regulation after transcription [86, 87]. It is known that miR-208a and miR-195 promote hypertrophic growth in cardiac hypertrophy by targeting signaling pathways that regulate cardiomyocyte size. Conversely, miR-133 and miR-1 act as anti-hypertrophic miRNAs by inhibiting the synthesis of genes associated with hypertrophy [102]. In myocardial fibrosis, miR-21 and miR-29 also have opposing roles. MiR-21 causes fibrosis by targeting phosphatase and tensin homolog (PTEN) and promoting fibroblast proliferation, whereas miR-29 functions as an anti-fibrotic regulator by preventing the synthesis of collagen and extracellular matrix proteins [103]. This comparative study emphasizes the dual roles of miRNAs as pro- or anti-regulators, highlighting their potential as therapeutic targets in cardiac hypertrophy and fibrosis [104]. RNA modification, which impacts a variety of RNA types, is a critical component of post transcriptional control. More recently, RNA alterations have been recognized as important factors in the prediction and treatment of disease risk, surpassing the constraints of DNA sequence analysis. RNA modifications are crucial in many biological processes as well as human conditions because they have a major impact on RNA translation, stability, and transfer [105, 106].

Cardiac hypertrophy is one of the main ways the heart compensates for a range of pathological stresses. The response's primary objectives are to control wall stress and preserve contractile function, but over time, it leads to hypertrophy and may eventually induce heart failure. Although several routes work together to coordinate the hypertrophy process, little is known about the molecular mechanisms behind these pathways [107]. Cardiac remodeling brought on by specific stressors may have detrimental effects and change the gene expression profile [108]. This degenerative process is now recognized to be influenced by miRNAs, and there is mounting evidence that miRNAs are also important regulators of cardiovascular development and function, and ventricular hypertrophy. Of the numerous miRNAs that have been shown to be altered during this condition, miR-1 and miR-133 are essential in preventing cardiac hypertrophy [109, 110]. MiR-1 and miR-133 are members of the same bicistronic unit and are specifically expressed in cardiac myocytes and skeletal muscle. *In vivo*, embryonic overexpression of miR-1 results in thin-walled ventricles, whereas miR-1 knockout mice have thicker-walled chambers. The changes in gene expression that underpin the onset and progression of ventricular hypertrophy appear to be sufficiently explained by the down-regulation of MiR-1 at the onset of cardiac pressure overload. MiR-1 suppresses calcium-calmodulin signaling through the calcineurin/NFAT pathway and negatively regulates Mef2a and Gata4 expression to stop cardiomyocyte growth [111]. Twinfilin-1, a cytoskeleton regulating protein, is one of miR-1's novel targets. When hypertrophic stimuli reduce miR-1, twinfilin-1 is up-regulated, which triggers hypertrophy by controlling the cardiac cytoskeleton [112, 113].

Heart failure, dilated and hypertrophic cardiomyopathies, and myocardial infarction are among the heart disorders that exhibit cardiac fibrosis, a well-known morphological feature of structural myocardial remodeling [114]. The fundamental basis of fibrosis is the accumulation of collagens and other extracellular matrix (ECM) proteins, which increases the risk of dangerous cardiovascular events such as ventricular failure and arrhythmias [115]. A few studies have demonstrated that ischemia or mechanical stress is associated with altered expression of various miRNAs in myocardial fibrosis, however the precise cause of the fibrogenic cardiac phenotype remains uncertain. MiR-29 controls cardiac fibrosis39 and is also a possible therapeutic target for tissue fibrosis in general. The miR-29 family targets several mRNAs that encode proteins linked to fibrosis, such as several collagens, fibrillins, and elastins [116, 117]. Consequently, it is expected that downregulating miR-29 will reduce the synthesis of certain mRNAs and enhance the fibrotic response. Collagen expression is actually increased when miR-29 is down-regulated *in vitro* and *in vivo*, whereas it is decreased when miR-29 is overexpressed in fibroblasts39. It was shown that miR-2939 down-regulation was accompanied by down-regulation of miR-149 and up-regulation of miR-21, 214, and 223 [118].

Non-coding RNA is a class of RNA molecules that control cellular function and disease development but do not make proteins. It has garnered significant attention due to recent developments in the biology of cardiovascular disorders. Long-stranded RNA, short-stranded RNA (like small molecule RNA), and other RNAs fall within this wide group [119]. Long non-coding RNAs, or LncRNAs, are RNA blocks of 200 bases or more. They have proven to be quite beneficial in managing heart disease. One such condition that is caused by a long noncoding RNA generated by the Tyk2-ROG1 gene is heart valve disease. This RNA negatively regulates pathways associated with inflammation [120]. Additionally, it has been suggested that the heart enlarges and ultimately fails as a consequence of excessive synthesis of Xist lncRNA in cardiomyocytes, which disrupts the balance of X-chromosome inactivation and interferes with the expression of genes critical for cardiac function, leading to cardiac failure [121]. Vital genes on the active X-chromosome are abnormally silenced as a result of cardiomyocytes' overproduction of Xist lncRNA, which upsets the balance of X-chromosome inactivation. This imbalance affects genes necessary for calcium signaling, mitochondrial function, and sarcomeric integrity, thereby impairing cardiomyocyte contractility and energy metabolism. The resulting cellular stress leads to maladaptive hypertrophic signaling pathways and fibrosis, which ultimately result in pathological heart hypertrophy and failure [122, 123].

Consequently, Xist offers a promising new opportunity for the treatment of cardiovascular disease. The small size of short non-coding RNAs, including lncRNAs, siRNAs, and miRNAs, is one of their distinguishing features. The well-known miRNA miR-1 has a variety of roles in heart conditions. Its expression levels directly affect cardiac function, and studies suggest that by controlling cardiac structure and preventing cell division and proliferation, it may lessen the harmful effects of cardio-myopathy [124]. On the other hand, miRNA-223 increases endothelial cell death and cardiac hypertrophy, which affects the incidence of atrial fibrillation [125]. Because long noncoding RNAs (lncRNAs) are microscopic regulators of gene expression that control a multitude of processes, including cell proliferation, death, and heart remodeling, they are important players in the development of cardiovascular disease [126, 127]. Many non-coding RNAs are part of a complex regulatory network and have varied roles in cardiovascular disease. Because of this, a careful analysis of their molecular mechanisms and functional traits may identify new therapeutic targets and diagnostic markers, significantly adding to our understanding of the treatment and prevention of cardiovascular disease [128, 129]. Notable earlier findings in the area of cardiovascular disease epigenetics are included in Table **2**.

## 3 HISTORICAL DEVELOPMENT OF EPIGENETIC MECHANISMS IN HEART HYPERTROPHY AND FIBROTIC SCARRING

The English scientist C.H. Waddington coined the term “epigenetics”, which he used to describe “changes in non-genetic sequences”, in 1942. Based on the well acknowledged idea that DNA methylation may change gene activity, British molecular biologist R Holliday did not systematically redefine the word “epigenetic” in an academic study until the 1980s. According to Holliday (1990), epigenetics is the study of the spatial and temporal processes by which genes activate throughout the development of complex animals [130]. In 1996, American geneticist Athur D. Riggs and his associates defined epigenetics as the genetic changes in gene function caused by meiosis or mitosis that do not change the genetic sequence. S.A. Bird, a British scientist, defined epigenetics as the structural alteration of a chromosome that causes it to express itself, emit signals, or maintain a modified state of activity in 2007 [131].

At a Cold Spring Harbor conference in 2008, the core of epigenetics was defined as chromosomal modifications that produced enduring hereditary traits without a change in the DNA sequence. Additionally, in 2013, the National Institutes of Health (NIH) in the United States stated that epigenetics included both hereditary changes in gene activity and expression in cells or individuals as well as stable, long-term, and non-inherited alterations at the potential level of cell transcription [132]. According to the currently recognized theory of epigenetics, alterations in DNA sequence do not cause heritable changes in gene expression. Through covalent changes to histone proteins and nucleic acids that together regulate chromatin structure, epigenetic mechanisms have been found and are widely accepted because they regulate gene expression without changing the DNA sequence [133]. Reversible epigenetic regulators continuously regulate gene expression. This implies that the role of epigenetic mechanisms in biology could be becoming more and more important. It also enables the creation of epigenetic drugs to treat particular diseases [134]. Table **2** lists some significant historical developments in cardiovascular disease epigenetics.

### 3.1 Epigenetic Mechanisms Driving Cardiac Hypertrophy

Cardiac hypertrophy is the enlargement of heart muscle cells, often as an adaptive response to an increased workload or stress. However, chronic hypertrophy can lead to heart failure. Key epigenetic factors involved include:

#### 3.1.1 DNA Methylation

- Alterations in DNA methylation patterns have been associated with cardiac hypertrophy. For example, hypermethylation of certain gene promoters can silence genes that normally inhibit hypertrophy.
- A study by some researchers demonstrated that DNA methyltransferase 3B (DNMT3B) contributes to hypertrophic growth by methylating genes involved in cardiac stress responses [135]. Fig. (**1**) illustrates the previous advancements in the field of cardiovascular disease epigenetics.

#### 3.1.2 Modifications in Histone

- Histone acetylation and methylation play roles in regulating hypertrophic gene expression.
- Histone acetyltransferases (HATs) and deacetylases (HDACs) modify histones to either promote or inhibit transcription. For example, HDAC inhibitors have shown potential in reversing hypertrophic growth by allowing the expression of anti-hypertrophic genes [136].

#### 3.1.3 Non-coding RNAs

miRNAs, particularly miR-208, have been shown to modulate hypertrophic pathways. Inhibition of specific miRNAs can attenuate hypertrophy, suggesting potential therapeutic targets [137].

### 3.2 Epigenetic Pathways in Fibrotic Scarring

Fibrotic scarring, or cardiac fibrosis, involves excessive deposition of extracellular matrix components, leading to the stiffening of heart tissue. This condition often accompanies cardiac hypertrophy and contributes to heart dysfunction. Epigenetic factors include:

### 3.3 DNA Methylation

- Fibrotic gene expression is regulated by DNA methylation. For instance, hypomethylation of the collagen gene promoters can lead to increased collagen production, a hallmark of fibrosis.
- Recent studies have identified specific methylation signatures associated with fibrotic genes in cardiac tissue, offering insights into potential biomarkers for the early detection.

#### 3.3.1 Modifications in Histone

- Changes in histone marks, such as H3K9 and H3K27 methylation, have been linked to fibrotic gene regulation.
- Epigenetic therapies targeting histone modifications are being explored to prevent or reverse fibrosis. Inhibitors of histone methyltransferases have shown promise in reducing fibrotic gene expression.

#### 3.3.2 Non-coding RNAs

- miRNAs, such as miR-21 and miR-29, are crucial regulators of fibrosis-related pathways.
- Modulation of these miRNAs can alter fibrotic responses, presenting opportunities for therapeutic intervention [138].

### 3.4 Perturbations in Epigenetic Processes and their Impacts

Epigenetic perturbations refer to abnormal changes in these mechanisms, often induced by environmental factors, genetic predispositions, or pathological conditions. In cardiac hypertrophy and fibrosis, perturbations can manifest as:

#### 3.4.1 Aberrant DNA Methylation

- Environmental stressors, such as hypertension or diabetes, can lead to aberrant DNA methylation, driving maladaptive cardiac remodeling.

#### 3.4.2 Dysregulated Modifications in Histone

- Chronic stress can alter histone modification patterns, sustaining pathological gene expression and promoting disease progression.

#### 3.4.3 Altered Non-coding RNA Profiles

- Dysregulation of miRNAs and other non-coding RNAs can exacerbate hypertrophic and fibrotic signaling pathways, contributing to disease severity [139].

### 3.5 Clinical Implications and Therapeutic Approaches

Understanding epigenetic perturbations offers potential avenues for therapeutic intervention:

#### 3.5.1 Epigenetic Drugs

##### 3.5.1.1 DNA Methylation Inhibitors

Drugs like 5-azacytidine can reverse hypermethylation, potentially restoring normal gene function in cardiac cells.

##### 3.5.1.2 HDAC Inhibitors

Compounds such as vorinostat have shown efficacy in preclinical models by modulating histone acetylation.

#### 3.5.2 miRNA-based Therapies

Therapeutic miRNA mimics or inhibitors can correct dysregulated pathways. For instance, targeting miR-21 has been shown to reduce fibrosis in animal models [140].

#### 3.5.3 Personalized Medicine

Epigenetic profiling can identify individuals at risk of cardiac hypertrophy and fibrosis, allowing for tailored prevention and treatment strategies. Fig. (**2**) illustrates the fundamental mechanisms by which epigenetic and transcriptional modifications occur in cardiovascular disorders.

## 4 EPIGENETIC REGULATION

Epigenetic regulatory mechanisms are critical processes that influence the expression of genes without altering the underlying DNA sequence. These mechanisms are fundamental in various biological processes, including development, differentiation, and the pathogenesis of diseases such as cardiac hypertrophy and fibrotic scarring. Here, we'll explore the primary epigenetic regulatory mechanisms, their functions, and their roles in cardiac pathology [141].

Epigenetic regulation mechanisms involve several key processes:

- DNA methylation
- Modifications in histone
- Chromatin remodeling
- Non-coding RNAs
- Epigenetic memory and transgenerational epigenetics

Let's delve into each of these mechanisms and understand how they contribute to gene regulation and cardiac diseases like hypertrophy and fibrosis.

### 4.1 DNA Methylation

When DNA is methylated, 5-methylcytosine is created by adding a methyl group (CH_3_) to the 5-carbon cytosine residues, which are often found in CpG dinucleotides. This modification often acts as a mechanism for gene silencing [142]. DNA methylation regulates gene expression by appending methyl groups to CpG sites; its dysregulation is linked to cardiac diseases. The study of DNA methylation has been fundamentally altered by recent advancements that offer high-resolution and real-time insights, such as whole-genome bisulfite sequencing (WGBS) and nanopore-based methylome analysis [143]. Accurate functional studies and insight into the cell-specific epigenetic dynamics of cardiac hypertrophy and fibrosis are made possible by new technologies like single-cell methyl omics and CRISPR-dCas9 for targeted methylation editing [144]. A comprehensive picture of the epigenetic environment may be obtained by combining these methods with transcriptomics and proteomics. Some analyses of epigenetic regulation of DNA methylation are:

#### 4.1.1 Emerging Technologies

##### 4.1.1.1 Bisulfite Sequencing

Mention advancements in whole-genome bisulfite sequencing (WGBS), which provides single-base resolution and is widely used for mapping methylation patterns.

##### 4.1.1.2 Nanopore Sequencing

Highlight how nanopore sequencing enables real-time, long-read DNA methylation analysis without the need for chemical conversion, preserving DNA integrity.

##### 4.1.1.3 Methylation Arrays

Discuss the use of advanced methylation arrays like the Infinium Methylation EPIC BeadChip for high-throughput methylome analysis [145].

#### 4.1.2 Improved Methods

##### 4.1.2.1 Single-cell Methylomics

Explain the emergence of single-cell methylome sequencing techniques, which allow the study of cell-specific methylation dynamics.

##### 4.1.2.2 CRISPR-based Tools

Introduce CRISPR-dCas9 systems for targeted DNA methylation editing, which enable functional validation of specific methylation sites.

#### 4.1.3 Technological Integration

##### 4.1.3.1 Multi-omics Approaches

Discuss combining methylation data with transcriptomics, proteomics, and histone modification data to gain holistic insights into epigenetic regulation [146, 147].

#### 4.1.4 Key Enzymes

- DNMT1 (DNA Methyltransferases): DNMT1 keeps the methylation patterns in place when DNA replicates.
- DNMT3A/B - Establishes new methylation marks during development.

##### 4.1.4.1 Ten-eleven Translocation (TET) Enzymes

TET enzymes convert 5-methylcytosine to 5-hydroxymethylcytosine, which can lead to demethylation [148].

#### 4.1.5 Mechanism

##### 4.1.5.1 CpG Islands

Genome regions with a high frequency of CpG sites. Methylation of CpG islands, often found in promoter regions, can repress gene expression by inhibiting the binding of transcription factors.

##### 4.1.5.2 Gene Silencing

Methylation can lead to chromatin compaction, preventing transcriptional machinery access.

#### 4.1.6 Role in Cardiac Pathology

##### 4.1.6.1 Cardiac Hypertrophy

Aberrant DNA methylation patterns can lead to inappropriate expression of genes involved in hypertrophy, contributing to pathological heart enlargement.

##### 4.1.6.2 Fibrotic Scarring

Hypomethylation of fibrosis-related genes may enhance fibroblast activation and extracellular matrix production, exacerbating fibrosis.

### 4.2 Modifications in Histone

Histone changes are caused by covalent post-translational modifications (PTMs) to histone proteins. These modifications affect chromatin structure and gene expression. Common modifications include phosphorylation, ubiquitination, sumoylation, acetylation, and methylation [149].

#### 4.2.1 Key Enzymes

##### 4.2.1.1 Histone Acetyltransferases (HATs)

Generally associated with the expression of genes, HATs add acetyl groups to lysine residues.

##### 4.2.1.2 Histone Deacetylases (HDACs)

Deletion of acetyl groups by histone deacetylases (HDACs) often results in the silencing of genes.

##### 4.2.1.3 Histone Methyltransferases (HMTs)

Depending on where they are added, methyl groups added to arginine or lysine residues may either increase or decrease the expression of a gene.

##### 4.2.1.4 Histone Demethylases (HDMs)

The removal of methyl groups by histone demethylases (HDMs) modifies the chromatin state and the activity of genes.

#### 4.2.2 Mechanism

##### 4.2.2.1 Acetylation

Neutralizes the positive charge of histones, decreases affinity for negatively charged DNA and promotes an open structure of chromatin conducive to transcription.

##### 4.2.2.2 Methylation

The effect depends on the specific amino acid residue and the number of methyl groups added [150].

**For example:** H3K4me3 is usually a mark of active promoters. H3K9me3 and H3K27me3 are typically associated with repressed chromatin.

#### 4.2.3 Role in Cardiac Pathology

##### 4.2.3.1 Cardiac Hypertrophy

Modifications in histones can alter the expression of genes that regulate cardiac muscle cell growth and stress response, contributing to hypertrophy.

##### 4.2.3.2 Fibrosis

Changes in histone marks can promote the transcription of profibrotic genes, facilitating fibroblast activation and matrix deposition.

### 4.3 Chromatin Remodeling

The term “chromatin remodeling” describes the dynamic rearrangement of chromatin to allow packaged genomic DNA to be accessed by regulatory transcription machinery proteins, which in turn regulates the expression of genes [151].

#### 4.3.1 Key Complexes

##### 4.3.1.1 SWI/SNF

Uses ATP to slide or evict nucleosomes, facilitating access to DNA. Nucleosome eviction and sliding are the two primary ways whereby SWI/SNF complexes modify chromatin structure by hydrolyzing ATP [152]. The sliding mechanism makes regulatory regions visible and facilitates transcriptional activation or repression by moving nucleosomes along the DNA. Conversely, nucleosome eviction occurs when histone octamers are completely removed from DNA, leaving nucleosome-free regions. This eviction mechanism is necessary for processes that require access to the DNA template, such as transcription initiation, which requires open chromatin, and DNA damage repair. Studies have shown that coordinated interactions with histone chaperones are necessary to ensure proper histone removal and recycling during nucleosome eviction by SWI/SNF [153]. The ATP-dependent chromatin remodeling complex SWI/SNF regulates transcription. Using energy from ATP hydrolysis, these complexes transport nucleosomes, remove or rebuild histones, and encourage the exchange of histone variants. Nucleosome eviction is necessary for processes that require access to the DNA template, such as transcription initiation and DNA damage repair. Conversely, access remodelers boost gene expression by permitting transcription factors to enter the chromatin [154, 155].

##### 4.3.1.2 ISWI, CHD, INO80

These complexes have distinct roles in altering nucleosome positioning, assembly, and stability.

#### 4.3.2 Mechanism

##### 4.3.2.1 Nucleosome Sliding

The repositioning of nucleosomes to expose or obscure DNA regions.

##### 4.3.2.2 Histone Exchange

Replacement of canonical histones with histone variants, altering chromatin properties.

##### 4.3.2.3 DNA Accessibility

Changes in nucleosome positioning affect the DNA-binding ability of transcription factors and regulate the expression of genes.

#### 4.3.3 Role in Cardiac Pathology

##### 4.3.3.1 Cardiac Hypertrophy

Remodeling complexes can alter chromatin structure around hypertrophic genes, leading to their activation.

##### 4.3.3.2 Fibrosis

Remodeling can facilitate transcription of extracellular matrix production involved genes and fibroblast proliferation.

### 4.4 Non-coding RNAs

Non-coding RNAs are RNA molecules that are not translated into proteins but have a significant impact on transcriptional and post-transcriptional regulation of gene expression. These consist of small interfering RNAs (siRNAs), long non-coding RNAs (lncRNAs), and microRNAs (miRNAs) [156].

#### 4.4.1 Types and Functions

##### 4.4.1.1 MicroRNAs (miRNAs)

Small, ~22 nucleotides long, they regulate gene expression by binding to complementary sequences in mRNA, leading to mRNA degradation or translational repression.

##### 4.4.1.2 Long Non-coding RNAs (lncRNAs)

Typically longer than 200 nucleotides, they can regulate gene expression through diverse mechanisms, including remodeling of chromatin, transcriptional interference, and post-transcriptional control.

##### 4.4.1.3 Small Interfering RNAs (siRNAs)

Involved in RNA interference, they can silence gene expression by targeting mRNA for degradation.

#### 4.4.2 Mechanism

##### 4.4.2.1 miRNA-mediated Regulation

miRNAs bind to target mRNA 3' UTRs, leading to either degradation or inhibition of translation.

##### 4.4.2.2 lncRNA Interaction

lncRNAs can interact with DNA, RNA, or proteins to modulate gene expression. They can act as scaffolds, guides, or decoys for regulatory proteins [157].

#### 4.4.3 Role in Cardiac Pathology

##### 4.4.3.1 Cardiac Hypertrophy

Dysregulated miRNAs can affect the expression of genes involved in cardiac growth and response to stress.

##### 4.4.3.2 Fibrosis

Specific miRNAs and lncRNAs can modulate the activity of profibrotic pathways, influencing fibroblast behavior and matrix deposition [158].

### 4.5 Epigenetic Memory and Transgenerational Epigenetics

Epigenetic memory refers to the stable transmission of epigenetic marks across cell divisions. Transgenerational epigenetics involves the inheritance of epigenetic changes from one generation to the next, potentially affecting offspring without altering DNA sequences.

#### 4.5.1 Mechanism

##### 4.5.1.1 Epigenetic Inheritance

Epigenetic marks such as DNA methylation and modifications in histone can be preserved during cell division and, in some cases, passed through germ cells to offspring.

##### 4.5.1.2 Environmental Influences

Environmental factors, including diet, stress, and toxins, can induce epigenetic changes that may be heritable.

#### 4.5.2 Role in Cardiac Pathology

##### 4.5.2.1 Disease Susceptibility

Epigenetic memory can influence susceptibility to cardiac diseases, with inherited epigenetic marks predisposing individuals to conditions like hypertrophy and fibrosis. In the context of cardiac hypertrophy and fibrosis, these mechanisms play a pivotal role in mediating pathological changes, offering potential therapeutic targets for intervention [159]. An overview of genetic alterations in cardiovascular diseases is shown in Table **3** [141, 160-172].

### 4.6 Potential Therapeutic Approaches

#### 4.6.1 Epigenetic Drugs

Compounds targeting specific epigenetic modifications, such as DNMT inhibitors, HDAC inhibitors, or bromodomain inhibitors, are being explored for their potential to reverse pathological epigenetic changes.

Compounds that target certain epigenetic alterations, such as DNA methylation, histone acetylation, and chromatin remodeling, are being researched as a means of reversing harmful epigenetic changes. For instance, HDAC inhibitors like vorinostat, romidepsin, and panobinostat are used in cancer therapy to restore normal acetylation levels, which encourage gene re-expression and apoptosis, while DNMT inhibitors like azacitidine and decitabine have been approved to treat myelodysplastic syndromes and acute myeloid leukemia by reactivating silenced tumor suppressor genes [173]. Novel compounds that target BET proteins, such as bromodomain inhibitors (*e.g.*, JQ1), disrupt the link between transcriptional machinery and acetylated histones and may be useful in malignant and inflammatory diseases. Moreover, new medications called EZH2 inhibitors (tazemetostat, for instance) target histone methylation processes associated with aggressive cancers. In addition to showing the therapeutic promise of epigenetic control in cancer, these drugs are being studied for use in autoimmune disorders, neurological diseases, and cardiac fibrosis [174].

##### 4.6.1.1 miRNA-based Therapies

Modulating miRNA activity through mimics or inhibitors may offer new avenues for treating cardiac hypertrophy and fibrosis.

##### 4.6.1.2 Gene Editing

Technologies like CRISPR-Cas9 are being investigated for their ability to directly modify epigenetic marks or edit regulatory regions to correct dysregulated gene expression [175].

Epigenetic regulatory mechanisms are fundamental in the regulation of gene expression, impacting various biological processes and diseases. In cardiac hypertrophy and fibrosis, understanding these mechanisms provides crucial insights into disease pathogenesis and opens up novel therapeutic possibilities aimed at modulating epigenetic states to improve cardiac health [176]. Fig. (**3**) illustrates cardiac scarring and the activation of cardiac fibroblasts. Table **4** lists the limited clinical trials on epigenetic drugs from the https://clinicaltrials.gov website that used an observational and/or experimental study methodology.

In preclinical research, epigenetic drugs have demonstrated potential in reducing cardiac hypertrophy and fibrotic scarring. HDAC inhibitors such as trichostatin A and valproic acid have been shown to successfully reduce hypertrophic markers such as ANP and BNP in rat models. When compared to untreated controls, studies have shown a significant decrease in left ventricular wall thickness (by around 30%) and fibrosis area (by roughly 25%) [177]. In contrast, DNMT inhibitors like decitabine have reversed pathological DNA hypermethylation patterns, restored normal expression levels of antifibrotic genes like PPARγ, and significantly reduced collagen deposition (up to 40%) in mouse models of cardiac fibrosis. JQ1 and other bromodomain inhibitors have also shown a 50% reduction in fibroblast activation and inflammatory cytokine production in preclinical studies [178, 179].

## 5 LITERATURE REVIEW

### 5.1 Role of DNA Methylation in Cardiac Hypertrophy

Meder *et al.* (2017) explore genome-wide DNA methylation changes in human heart failure, identifying specific methylation patterns linked to cardiac hypertrophy and providing potential biomarkers for disease prediction [29].

Gilsbach *et al.* (2014) detail how dynamic DNA methylation contributes to the regulation of cardiomyocyte function and disease, emphasizing the epigenetic landscape in cardiac hypertrophy [30].

Heymans *et al.* (2019) discuss how chronic pressure overload leads to heart failure through changes in DNA methylation and highlight potential therapeutic targets for reversing hypertrophy [180].

- **Modifications in histones:** The study identified increased histone acetylation at the promoters of pro-hypertrophic genes, suggesting a mechanism by which these genes become upregulated in hypertrophy. Additionally, histone deacetylase (HDAC) inhibitors were shown to reverse some of these changes.
- **DNA methylation:** Differential DNA methylation patterns were observed in genes regulating cardiac structure and function. These changes were correlated with the severity and progression of the disease.

### 5.2 Modifications in Histone in Cardiac Hypertrophy and Fibrosis

Papait *et al.* (2013) explore the complex dynamics of histone methylation in the heart under stress, providing insights into the regulatory mechanisms of cardiac hypertrophy and fibrosis [181].

- **Methylation States:** The study found that stress-induced changes in H3K4 methylation states lead to distinct gene expression profiles in cardiac cells. Trimethylation was associated with active transcription of hypertrophy-related genes, whereas monomethylation was linked to gene repression.
- **Epigenetic Enzymes:** The enzymes responsible for adding or removing these methylation marks, such as methyltransferases and demethylases, were identified as key regulators of the hypertrophic response.

Hohl *et al.* (2013) emphasize the role of histone acetylation and HDAC inhibition in controlling the hypertrophic response, suggesting potential therapeutic applications for managing cardiac hypertrophy [182].

Haberland (2009) have reviewed the multifaceted roles of histone deacetylases in development and their potential as therapeutic targets for diseases like cardiac hypertrophy and fibrosis [183].

### 5.3 MicroRNAs in Cardiac Hypertrophy and Fibrosis

Thum *et al.* (2008) highlight the role of microRNA-21 in promoting myocardial fibrosis and hypertrophy by activating MAP kinase signaling, demonstrating a direct link between miRNA and cardiac remodeling [184].

Kumarswamy and Thum (2013) have provided a comprehensive review and focused on non-coding RNAs, including microRNAs, and their regulatory roles in cardiac remodeling and heart failure, providing a broader context for therapeutic interventions [185].

Van Rooij *et al.* (2006) identify a specific pattern of stress-responsive microRNAs involved in cardiac hypertrophy and heart failure, paving the way for potential miRNA-based therapies [186].

### 5.4 Epigenetic Perturbations and Environmental Influences

Movassagh *et al.* (2011) demonstrate how environmental factors lead to differential DNA methylation in heart failure, affecting the expression of genes involved in angiogenesis and cardiac remodeling [187].

Greco and Condorelli (2015) discuss how external stressors and genetic predispositions cause epigenetic changes that contribute to cardiac hypertrophy and failure, emphasizing the importance of epigenetic regulation in disease progression [188].

Baccarelli *et al.* (2010) provide a detailed overview of cardiovascular epigenetics, highlighting how environmental factors can cause epigenetic perturbations leading to cardiac diseases [189].

### 5.5 Therapeutic Approaches and Future Directions

Zhang *et al.* (2019) review the current and emerging epigenetic therapies, focusing on histone acetylation and DNA methylation, and their potential to mitigate heart failure progression [190].

Dal-Pra *et al.* (2019) have explored various epigenetic therapies aimed at cardiac regeneration, discussing the challenges and opportunities in translating these therapies from bench to bedside [191].

Baylin and Jones (2016) provide insights into the evolution of epigenetic therapies, emphasizing their clinical potential in treating complex diseases, including cardiac hypertrophy and fibrosis [192].

Esteller M. (2008) focused on cancer; this seminal paper outlines key concepts in epigenetic therapy, offering parallels and insights applicable to cardiac conditions [193].

### 5.6 Combining Omics for Comprehensive Insights

Barth *et al.* (2006) integrate transcriptomic and proteomic data to provide a holistic view of the molecular changes in cardiac hypertrophy, offering a platform for identifying novel therapeutic targets [194].

Dweck and Boon (2012) highlight the role of advanced imaging techniques in understanding cardiac diseases, supporting the integration of imaging with molecular studies for a comprehensive approach [195].

Eschert H. *et al.* (2019) discuss the integration of various omics approaches in cardiovascular research, emphasizing their potential to unravel the complexities of cardiac hypertrophy and fibrosis [196].

### 5.7 Novel Epigenetic Biomarkers

Matkovich *et al.* (2014) have identified epigenetic biomarkers that correlate with cardiac hypertrophy, providing potential targets for early diagnosis and personalized treatment strategies [197].

Morrison *et al.* (2015) explore epigenetic biomarkers associated with atherosclerosis, offering insights into shared pathways in cardiac hypertrophy and fibrosis [198].

Kumarswamy *et al.* (2014) review and highlight circulating miRNAs as potential non-invasive biomarkers for cardiovascular diseases, including cardiac hypertrophy and fibrosis, and discuss their role in cell-cell communication [199].

### 5.8 Epigenetics in Cardiac Regeneration

Dimmeler and Leri (2008) explore the impact of aging and disease on the efficacy of cell therapy in cardiac regeneration, highlighting the role of epigenetic modifications in therapeutic outcomes [200].

Marbán (2018) discusses the potential of stem cell therapy in cardiac regeneration, emphasizing the role of epigenetic factors in enhancing therapeutic efficacy [201].

Takeuchi and Bruneau (2009) demonstrate the directed trans-differentiation of mesodermal cells into cardiac tissue, highlighting the importance of epigenetic regulation in cardiac regeneration [202].

Gilsbach (2014) explore the dynamic nature of DNA methylation during cardiomyocyte development and in the context of cardiac diseases like hypertrophy. The research highlights the role of DNA methylation in regulating gene expression throughout the life cycle of cardiomyocytes [203]. Fig. (**4**) illustrates the relationships between heart disease and epigenetics.

One of the most promising techniques for solving the information gaps in the inheritance of common features is epigenomics. The study of the processes that allow cells to connect their genes and environment and adapt quickly to changes in the environment is known as epigenetics. Individual variations in gene epigenetic modification may account for a higher fraction of human phenotypic variability than genotype variants alone. Numerous in-depth studies have explored the association between epigenetics and CVD risk factors, including homocysteine, the biomarker most significantly associated with epigenetic processes tied to CVD risk. By changing the DNA methylation of vascular smooth muscle cells, homocysteine plays a role in the atherogenesis process. According to Rayner *et al.*, Mir-33 is a regulator of cholesterol homeostasis that affects cellular cholesterol export and the liver's generation of HDL.The brain possesses hydroxymethyl 2' deoxycytidine, which may contribute to the epigenetic control of neuronal activity, even if there are currently no confirmed correlations between hydroxymethylcytosine residues and CVD.

## 6 FUTURE PROSPECTIVE AND DIRECTIONS

Over the last 20 years, a multitude of new epigenetic discoveries have propelled research into cardiovascular disease [204]. This small study is just the beginning of the benefits that epigenetic theories and tools have brought to the field of heart fibrosis research. Many of the items in this list are enzymes, the activity of which can be modified by small-molecule drugs for therapeutic effects. HDAC inhibitors, for instance, have been successfully used in the lab to treat certain cancer types. The increased knowledge about human functional genome supports the idea that human disorders are mediated by epigenetic regulation [205]. Considering this, further investigation into the epigenetic pathways behind heart fibrosis will eventually lead to a remedy for this crippling condition.

### 6.1 Biomarker Discovery

Further research is needed to identify reliable epigenetic biomarkers for the early diagnosis and prognosis of cardiac hypertrophy and fibrosis.

### 6.2 Mechanistic Studies

Understanding the precise molecular mechanisms by which epigenetic modifications influence cardiac pathology is crucial for developing targeted therapies [206].

### 6.3 Integration with Other Omics

Combining epigenetics with genomics, transcriptomics, and proteomics can provide a comprehensive understanding of cardiac diseases and help uncover novel therapeutic targets.

The progress in multi-omics technology has led to an increased understanding of the cellular and genetic processes behind cardiac fibrosis and CF inflammation. Thanks to single-cell RNA-seq technology, we have been able to deconvolute healthy and fibrotic hearts at a deep resolution, and we have found an unanticipated biochemical difference. As of right now, this technology is evolving and offering incredibly precise genetic data across several cells in space and time, as well as within a single cell. The incorporation of geographic context in these datasets will be necessary to comprehend changes in fibrotic niche and aberrant cellular crosstalk that accompany heart disease [207]. Regrettably, the current constraints of spatial transcriptomics arise from its incapacity to target a multitude of genes many of which need to be pre-selected in a panel and its tendency to prioritize extraordinarily abundant transcripts of the genes that are expressed more often. As a result, it will be possible to compare research more accurately and draw conclusions from the resulting data that are more reliable [208]. Finally, it is essential to keep in mind that the use of these technologies as diagnostic tools for quantifying cardiac fibrosis has a practical constraint since they require the invasive process of obtaining fresh heart tissue, which cannot be done on patients who are still alive. However, these technologies expedite our comprehension of detrimental cardiac alterations and aid in the discovery of new therapeutic targets for the control of cardiac fibrosis [209].

Consequently, small-molecule inhibitors have been recognized as promising treatment possibilities for a range of diseases, including cancer, as our understanding of epigenetic control has expanded. Numerous studies, many of which are included in this review, have shown the role that epigenetics plays in the development of cardiac fibrosis and the anti-fibrotic effects of different inhibitors, notably those that target the HDACs or BET proteins. However, due to two major unresolved issues, no clinical trial has been carried out to evaluate any of them for fibrotic diseases: uncertainty regarding specific mechanisms underlying epigenetic control of cardiac fibrosis and the possible adverse effects of pan-inhibitors on other tissues or cells [210]. Because epigenetic alterations are dynamic, the field of epigenetic medicine has shown great promise. The area of epigenetics research in fibrotic disorders is very new when compared to other illnesses. Different studies have shown different results for the same epigenetic drug depending on the animal type, dose, or fibrotic stage [211]. Consequently, further investigation is needed to precisely measure the reparative effects of epigenetic drugs and to understand the exact control of their anti-fibrotic actions after administration. By focusing on the catalytic activity of epigenetic factors rather than pan-inhibitors, these studies will aid in the creation of more targeted inhibitors.

They may also enable more effective clinical use of epigenetic treatment for fibrotic disease. Another important problem with epigenetic treatments that might have detrimental effects on other systems and cells is their lack of cell selectivity. The advancement of cell-specific delivery methods will provide more specialized responses [212]. The area of anti-fibrotic therapeutic investigations is greatly impacted by attempts to selectively target active CF cells for medication delivery, since these cells serve as the key trigger cells for the development of the disease. These consist of functionalized nanoparticles covered with viral vectors or fibroblast proteins. For instance, systemic delivery of a cardiotropic AAV vector (serotype 9), including a Postn promoter, has shown the advantages of specifically targeting activated CFs in a mouse model of MI [213].

In a mouse model of MI, transgenic T cells that developed a chimeric antigen receptor against the protein FAP were able to precisely target and eliminate damaging CFs, as shown further subsequently [214]. The development of tailored drugs that target the most fibrotic populations while limiting off-target effects is made feasible by the ability to distinguish multiple functional states among diverse fibroblast subpopulations using single-cell RNA-seq approaches. In conclusion, by leveraging the most recent advancements in omics technologies in conjunction with a deeper understanding of the epigenetic mechanisms that trigger the fibrotic response, it should be possible to diagnose heart fibrosis more accurately and develop rational, highly targeted anti-fibrotic therapies [215-219].

Table **5** lists some of the most recent clinical trials for primary and secondary prevention from https://clinicaltrials.gov website that used an observational and/or experimental study methodology. The majority of patients with cardiovascular diseases, such as atherosclerosis, coronary heart disease, heart failure, hypertension, myocardial infarction, and others, participated in these epigenetics-based clinical studies and related epigenetic medicine research. However, most of the studies looking at how epigenetic regulation is used in heart disease are still in the experimental or clinical stages. Even so, there are certain epigenetic drugs that don't work well when used to treat heart problems. In order to better address the symptoms and prognosis of patients with cardiovascular diseases, it is anticipated that more cutting-edge epigenetic drugs will be developed in the future through comprehensive clinical research and ongoing investigation.

## CONCLUSION

In conclusion, the epigenetic mechanisms such as enzyme-dependent rearrangement, histone changes, and DNA methylation are essential for the proper development, function, and response to cardiac stress in a healthy heart. Examining cardiac development with a view to understanding these complex processes might include considering well-defined temporo-spatial “checkpoints”. A thorough understanding of the role that epigenetic processes play in cardiac edema and fibrotic scarring may be acquired from the studies and reviews that were previously described. Epigenetic changes, such as DNA methylation, modifications of histones, and RNA-based processes, may lead to the development of disease, particularly cardiovascular disease. These changes serve as the biological basis for harmful environmental inputs. The majority of heart hypertrophy and fibrotic scarring is caused by epigenetic mechanisms. Deviations from these processes might lead to incorrect cardiac remodeling, which emphasizes the need for further study to completely understand the complex relationships at work. It is possible to reduce these disorders and improve patient outcomes by using the information from these articles to develop more personalized medicines that aim to improve heart function and reverse aberrant changes. The development of novel, efficient therapies for cardiac disease and regenerative medicine will depend heavily on this knowledge in the future.

## Figures and Tables

**Fig. (1):**
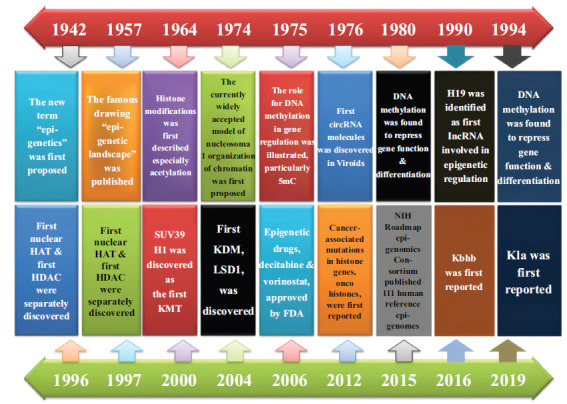
Significant previous advancements in the field of cardiovascular disease epigenetic.

**Fig. (2):**
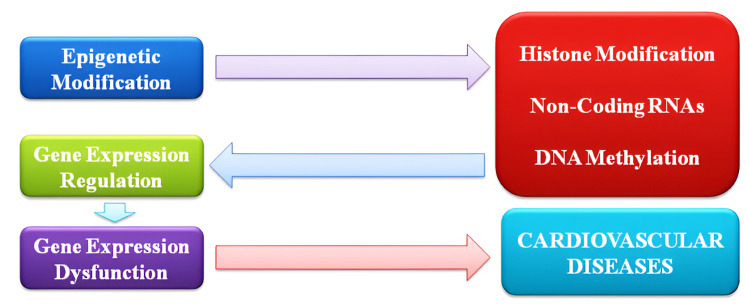
The fundamental mechanisms by which epigenetic and transcriptional modifications occur in cardiovascular disorders.

**Fig. (3):**
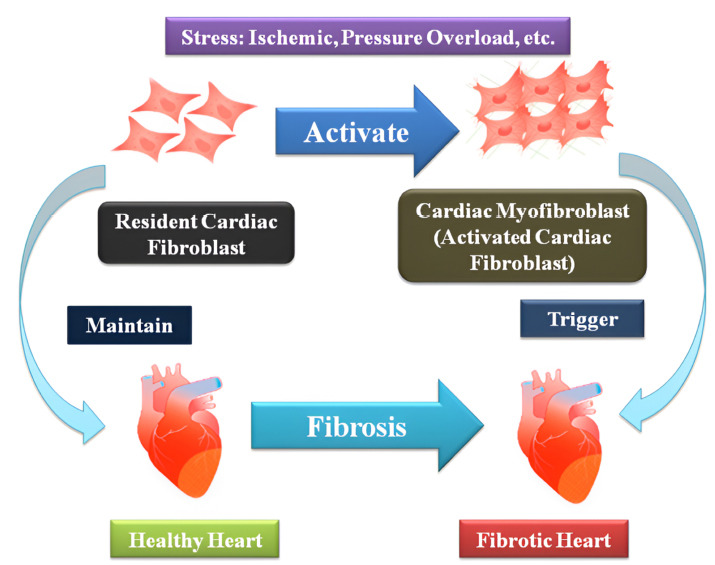
Cardiac scarring and activation of cardiac fibroblasts. The cellular and molecular activation of CFs into myofibroblasts is the cause of heart fibrosis. Fibroblasts found in the native heart help maintain the heart's balance and form. A variety of clinical and environmental stresses activate local cardiomyocytes (CFs) in the injured heart as myofibroblasts. These cells then produce and deposit an excessive amount of extracellular matrix (ECM), infiltrate the wounded area, and eventually result in cardiac fibrosis.

**Fig. (4):**
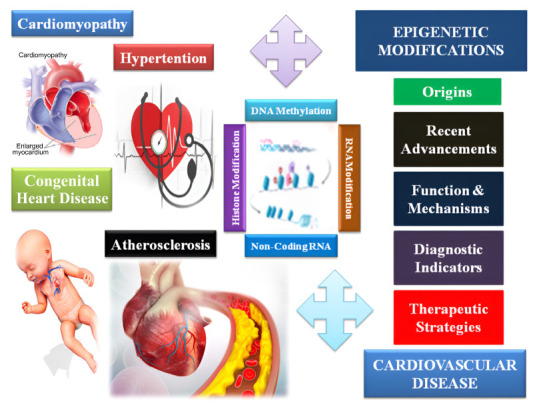
A summary of the relationships between heart disease and epigenetics.

**Table 1:** Transcriptional regulatory effects with epigenetic modifications.

**Mechanism**	**Transcriptional Effect**
**DNA-base Modifications**	
Methylation (CpG dinucleotides)	↓
Hydroxy-methylation (CpG dinucleotides)	↑
**Histone Code**	
Histone H3	↑
Acetylation (K9, K14)	↑
Methylation	↓
K4/9	↓
**Histone H4**	
Acetylation (K5, K8, K12, K16)	↑
Methylation (K20)	↓
**RNA-based Mechanisms**	
Repressive noncoding RNAs: Xist, ANRIL	↓
Activating noncoding RNAs: HOTTIP	↑

**Note:** HOXA distal transcript antisense RNA; K, lysine; S, serine; ↑, up; ↓, down; ANRIL, antisense noncoding RNA in INK4 gene.

**Table 2:** Significant historical developments in cardiovascular disease epigenetics.

**S. No.**	**Year**	**Developments in Cardiovascular Disease Epigenetics**
1	2005	It was first discovered that MCT3 DNA methylation silencing regulates development of atherosclerosis.
2	2006	Class I and II HDAC inhibitors (Scriptaid and Trichostatin) decrease pressure-overload cardiac hypertrophy, as was initially noted in circulation.
3	2011	Chemical inhibition of a miR-33a and miR-33b may improve cholesterol associated to risk of cardiovascular disease.
4	2014	The HDAC inhibitor SAHA was shown to elevate ischemia/reperfusion injury in circulation *via* inducing cardiac autophagy.
5	2016	A novel and effective medication for reducing Lp(A) levels in patients with cardiovascular disease is IONIS-APO(a)-LRx.
6	2017	The first clonal hematopoiesis and TET2 mutation accelerated atherosclerosis.
7	2018	The New England Journal discovered that in acute coronary patients’ syndrome, alirocumab enhanced cardiovascular outcomes after high-intensity statin therapy.
8	2019	The initial genetic risk locus for abdominal aortic calcification is HDAC9.
9	2020	Pulmonary arterial hypertension is first human illness linked to putative TET2 germline mutations.
10	2022	The epigenetic enzyme DOT1L prevents atherosclerosis by means of NF-κB pathway.

**Table 3:** An overview of research on genetic alterations in cardiovascular diseases.

**Diseases**	**Epigenetics Type**	**Major Regulator**	**Effect**	**References**
Congenital heart disease	DNA methylation	APOA5, PCSL9	Promote	[160]
	Histone modification	H3K4, H2BK120	Promote	[141]
	Non-coding RNAs	SOX4, HAS2, NF1	Inhibit	[161, 162]
Cardiomyopathy	DNA methylation	SERCA2a	Promote	[163]
	Histone modification	cTnl, ssTnl, HDAC1/2	Inhibit	[164]
	Non-coding RNAs	-	-	-
Hypertension	DNA methylation	11^®^-HSD-2, ECE-1, AT1b	Promote	[164]
	Histone modification	SIRT3, DRP1, SIRT6	Inhibit/ Promote	[165, 166]
	Non-coding RNAs	ACE2, CLIC4, ARF6	Promote	[167, 168]
Atherosclerosis	DNA methylation	VB6, MTHFR, LOX-1	Inhibit	[169, 170]
	Histone modification	HDAC1/2/3	Promote	[171]
	Non-coding RNAs	APOA, CXCL12	Promote	[172]

**Table 4:** Clinical trials on epigenetic drugs.

**NCT Number**	**Epigenetic Drug**	**Title**	**Effect of Epigenetic Drugs**
NCT01420926	Azacitidine (DNMT Inhibitor)	A Phase 3 Study of Azacitidine in Elderly Patients With Acute Myeloid Leukemia (AML)	Evaluating overall survival in elderly AML patients treated with azacitidine
NCT03404193	Decitabine (DNMT Inhibitor)	Decitabine Plus Venetoclax in Patients With AML or Myelodysplastic Syndromes	Assessing the safety and efficacy of combination therapy in hematologic malignancies
NCT00217412	Vorinostat (HDAC Inhibitor)	Vorinostat for Relapsed/Refractory Cutaneous T-Cell Lymphom	Testing vorinostat’s effect on T-cell lymphoma outcomes
NCT01023308	Panobinostat (HDAC Inhibitor)	Panobinostat in Combination With Bortezomib and Dexamethasone for Relapsed Multiple Myeloma	Studying efficacy in relapsed multiple myeloma.
NCT01960348	Romidepsin (HDAC Inhibitor)	Romidepsin and Azacitidine for Myelodysplastic Syndromes.	Evaluating the combination in treating MDS
NCT02601950	Tazemetostat (EZH2 Inhibitor)	A Study of Tazemetostat in Adults and Adolescents With Advanced Solid Tumors or Hematologic Malignancies	Investigating efficacy in cancers with EZH2 mutations
NCT00414306	Valproic Acid (HDAC Inhibitor)	Valproic Acid for the Treatment of Heart Failure	Exploring HDAC inhibition in heart failure management.
NCT00865969	Belinostat (HDAC Inhibitor)	Belinostat in Relapsed/Refractory Peripheral T-Cell Lymphoma	Assessing safety and efficacy in T-cell lymphoma
NCT02437136	Entinostat (HDAC Inhibitor)	Entinostat in Combination With Pembrolizumab in Advanced Solid Tumors	Testing the combination therapy’s efficacy in enhancing immune checkpoint inhibitors.

**Table 5:** Clinical trials using epigenetics-based therapies for heart disease.

**NCT Number**	**Disease**	**Study Type**	**Epidrug**	**No. of** **Participants**	**Epigenetic Modification**	**Status**
NCT03354156	Atherosclerosis	Observational	Statin	45	Methylation of Histone/DNA and Inhibitors of HDAC	Completed
NCT02597127	Atherosclerosis	Interventional	ALN-PCSSC	501	Synthesis of PCSK9	Phase 2
NCT04528004	Heart Failure	Interventional	Nicotinamide riboside	40	Histone acetylation and RNA Non-Coding	Early Phase 1
NCT03485092	Heart Failure	Interventional	Empagliflozin	105	DNA methylation	Phase 4
NCT04950569	Heart Failure	Interventional	Levosimendan	136	miR-660-3p, miR-665 and miR-1285-3p	Phase 4
NCT03360981	Coronary Artery Disease	Interventional	Incretins	150	Histone acetylation and RNA Non-Coding	Phase 4
NCT02226510	Coronary Artery Disease	Interventional	Metformin	68	Activators of HDAC	Phase 4
NCT01715714	Coronary Artery Disease	Interventional	Statin	2630	Methyltranferases DNA Inhibition	Phase 4
NCT05210725	Coronary Artery Disease	Interventional	Rivaroxaban	20	Histone acetylation and DNA methylation	Phase 4
NCT03825250	Coronary Artery Disease	Interventional	Sodium Valproate	122	Histone acetylation	Phase 2
NCT00616772	Coronary Artery Disease	Interventional	ABT-335, Atorvastatin	682	Methylation of Histone/DNA Inhibitors of HDAC	Phase 3
NCT02096991	Hypertension	Interventional	BisphenolA	60	DNA methylation	Completed
NCT01217307	Myocardial Infarction	Interventional	Metformin	380	Activators of HDAC	Phase 3
NCT01438723	Ischemic Heart Disease	Interventional	Metformin	100	Activators of HDAC	Phase 4
NCT03655704	Pulmonary Arterial Hypertension	Interventional	Apabetalone	7	RNA Non-Coding	Completed (Early Phase 1)
NCT02393768	Atherosclerosis	Observational		40	DNA methylation, Histone acetylation and RNA Non-Coding	Completed
NCT03546062	Heart Failure	Observational		30	miRNAs	Completed
NCT03462277	Coronary Artery Disease	Observational		200	DNA methylation/ Hydroxy-methylation, DNMTs and TET Enzyme Family	Completed
NCT02149316	Myocardial Ischemic Reperfusion Injury	Interventional		60	miR-133b, miR-208a	Completed
NCT01875484	Myocardial Infarction	Observational		1200	miR-126	Completed
NCT00924937	Myocardial Infarction	Interventional		1002	DNA methylation, Histone acetylation and RNA Non-Coding	Completed
NCT04371809	Acute Coronary Syndrome	Observational		100	DNA methylation	Completed
NCT04282434	Pulmonary Arterial Hypertension	Observational		30	DNA methylation	Completed

## LIST OF ABBREVIATIONS

BET: Bromodomain and Extra-Terminal motif
CFs: Cardiac Fibroblasts
CHD: Congenital Heart Disease
CVDs: Cardiovascular Diseases
DNA: Deoxyribonucleic Acid
DNMT: DNA Methyltransferase
ECM: Extracellular Matrix
HATs: Histone Acetyltransferases
HDACs: Histone Deacetylases
HDM: Histone Demethylases
HMTs: Histone Methyltransferases
LSDs: Lysine Specific Demethylases
MI: Myocardial Infarction
PMTs: Protein Lysine Methyltransferases
RNA: Ribonucleic Acid
SAH: S-Adenosyl-L-Homocysteine
SAM: S-Adenosylmethionine
TET: Ten-eleven Translocation Enzymes
TOF: Tetralogy Of Fallot
VSD: Ventricular Septal Defect
